# Neuroretinal structure changes in infantile nephropathic cystinosis

**DOI:** 10.1186/s13023-025-04018-2

**Published:** 2025-10-09

**Authors:** Leonie Franziska Keidel, Neringa Jurkute, Benedikt Schworm, Katharina Hohenfellner, Siegfried Priglinger, Axel Petzold, Claudia Priglinger

**Affiliations:** 1 Department of Ophthalmology, Ludwig-Maximilian University, Munich, Germany; 2 Department of Neuroophthalmology, Moorfields Eye Hospital NHS Foundation Trust, London, UK; 3 Institute of Ophthalmology, University College London, London, UK; 4 Department of Neuroophthalmology, The National Hospital for Neurology and Neurosurgery, London, UK; 5 Department of Pediatric Nephrology, RoMed Clinic Rosenheim, Rosenheim, Germany

**Keywords:** Infantile nephropathic cystinosis, Optic nerve, Spectral domain optical coherence tomography, OCT, Cystine crystals

## Abstract

**Background:**

The aim of this study was to investigate the neuroretinal structure of patients with the lysosomal storage disease cystinosis.

**Methods:**

In this retrospective cross-sectional analysis, optical coherence tomography (OCT) was used to measure the peripapillary retinal nerve fiber layer (pRNFL), the optic disc volumes, the prelaminar depth and the macular ganglion cell layer volumes (mGCL) in patients with genetically confirmed infantile nephropathic cystinosis. The same measurements were repeated in an age -and spherical equivalent (SE) matched, healthy control group.

**Results:**

The cystinosis group included 40 patients (40 eyes) with a mean age of 20.6 ± 8.6 years and a SE of 0.47 ± 1.85. The healthy control group consisted of 30 patients (30 eyes) with a mean age of 20.7 ± 12.5 years and a SE of 0.47 ± 1.29. A pronounced deposition of crystals in the optic disc was observed in all cystinosis cases. Cystine crystals follow the nerve fibers in a dense, pearl-string pattern. A significantly thicker pRNFL and a higher rate of positive prelaminar depth was evident in the cystinosis group (839.7 ± 151.0 μm vs. 775.7 ± 79.6 μm, *p* = 0.004). A significantly smaller mGCL volume was found in the cystinosis group as compared to normal controls (0.25 ± 0.03 mm³ vs. 0.35 ± 0.03 mm³, *p* = 0.036).

**Conclusions:**

Cystinosis leads to pronounced crystal accumulation in the optic disc in early stages of the disease. This accumulation occurs in concomitance with the well-described cystine crystal deposits in the cornea, which have previously been considered the foremost ocular sign of cystinosis. The pearl-string appearance of crystal deposition suggests a primarily glial localization. A significantly thicker pRNFL and a higher rate of positive prelaminar depth was observed in the OCT scans of cystinosis patients, explaining the clinical impression of a crowded optic disc. Additionally, retinal neurodegeneration was significant in patients with cystinosis if compared to healthy controls. The optic disc crowding may result from the dense deposition of cystine crystals in the optic nerve head and the GCL thinning could be due to metabolically induced ganglion cell atrophy. However, the exact reason for these changes remains to be elucidated.

## Background

Cystinosis is a rare, autosomal recessive lysosomal storage disorder caused by pathogenic variants in the *CTNS* gene, which codes for the lysosomal amino acid transporter cystinosin [1]. Dysfunction of cystinosin leads to an accumulation of cystine within the lysosome and thus potentially to the apoptosis of the cell [2]. Infantile nephropathic cystinosis is the primary cause of renal Fanconi syndrome in children, a disorder that disrupts the energy balance of the proximal renal tubule cells and typically manifests in childhood [3]. Renal abnormalities, as well as corneal cystine crystals, can be detected as early as 6–12 months of age [4]. The diagnosis is confirmed by molecular genetic testing and by determining cystine levels in the leukocytes. Cystinosis can partly be treated by cysteamine, a drug which binds to lysosomal cystine and converts it into cysteine-cysteamine disulfide that can be exported out of the lysosome via the lysine/arginine and the cysteine transporters, respectively [5]. Cysteamine is approved for systemic therapy in an oral form and can be applied in a soluble form as local therapy (eye drops) [6].

To date, authors describe the cornea as the foremost localization in the eye where cystine crystals can be detected in large numbers (“the window to cystinosis”) [4]. There is less awareness of concomitant posterior segment alterations. Recently the retinochoroidal cystine crystal score (RCCCS) was developed by our group, a score designed to grade the chorioretinal cystine crystal deposition and it could be shown that the RCCCS correlates with renal function parameters and cysteamine intake [7].

The aim of this study is to highlight for the first time the pronounced presence of cystine crystals in the optic nerve head, which upon spectral domain optical coherence tomography (SD-OCT)-imaging are already visible at an early stage in concomitance with the corneal cystine crystal deposition and before chorioretinal cystine crystal accumulation. Another goal of the study is to describe the morphology of the optic disc and neuroretina comprehensively and objectively in a large cohort of cystinosis patients with the help of SD-OCT. SD-OCT is a non-invasive imaging modality that generates cross-sectional scans of the retina and optic disc in a high histological resolution of up to 1–4 μm axial resolution [8]. Given that SD-OCT uses light in the near-infrared spectrum which does not induce photophobia it is particularly suitable for cystinosis patients, who suffer from scattering of the light due to corneal cystine crystal depositions [9, 10].

## Methods

This cross-sectional monocenter study recruited patients with infantile nephropathic cystinosis from the German Interdisciplinary Cystinosis Clinic Germany between 2018 and 2023 [11]; ethics committee approval for this study was by Ethikkommission der Bayerischen Landesärztekammer Identifier 23,004; Ethikkommission LMU Munich Identifier 25–0079. A normal control group, matched for age and spherical equivalent was recruited. Consent to use their data for analysis and scientific publication was obtained from all participating patients/their legal guardians. The ethics committee identifier for measurements of healthy controls was 19–799. All research and measurements followed the tenets of the Declaration of Helsinki. All ophthalmological examinations were carried out at the University Eye Hospital, Ludwig-Maximilian University, Munich, Germany.

The control subjects had to meet the following inclusion criteria: (i) spherical equivalent (SE) between + 3.0 and − 3.0 diopters; (ii) BCVA of 0.0 logMAR or better; and (iii) no diagnosed ocular diseases or structural alterations. To avoid intraindividual bias, only one eye was selected from each control subject and cystinosis patient.

### Ophthalmological examination

A comprehensive ophthalmological examination was performed. It included slit lamp biomicroscopy, dilated fundoscopy, Goldmann applanation tonometry and best-corrected visual acuity (BCVA) using the standard ETDRS chart at a testing distance of four meters. The data on the clinical quantification of cystine crystals in the cornea determined with the Gahl Corneal Cystine Crystal Score (CCCS) [4], were obtained from a previous publication of our research group.

## Spectral-Domain optical coherence tomography

An SD-OCT system (Spectralis^®^; Heidelberg Engineering GmbH, Heidelberg, Germany) was used for B-scan acquisition. The peripapillary retinal nerve fiber layer (pRNFL) was measured with activated eye tracker using ring scans around the optic nerve head (12°, resolution: 768 A-scans, 496 pixels [Z], 57 ≤ ART ≤ 100) or the most inner ring of a star-and-ring scan around the optic nerve (12°, resolution: 768 A-scans, 496 pixels [Z], 57 ≤ ART ≤ 100) [12]. The volume data for the ganglion cell layer (GCL) was calculated as a 3 mm diameter cylinder centered to the fovea derived from a macular volume scan (20° × 20° [5.9 × 5.9 mm], 49 horizontal B-scans, resolution: 512 pixels [X] × 496 pixels [Z], 18 ≤ ART ≤ 30) [13].

Optic disc volumetry was performed using a volume scan centered to the optic disc and measuring the total retinal volume (internal limiting membrane to Bruchs membrane) in the 3.45 mm ETDRS grid with the manufacturer’s software according to a previously described method [14]. Image optimization using the automatic real-time (ART) averaging algorithm (12 frames per B-scan) of the onboard manufacturer’s software was employed.

All scans were checked for segmentation errors by experienced graders (L.K., C.P.) and corrected manually when necessary.

**Fig. 1:**
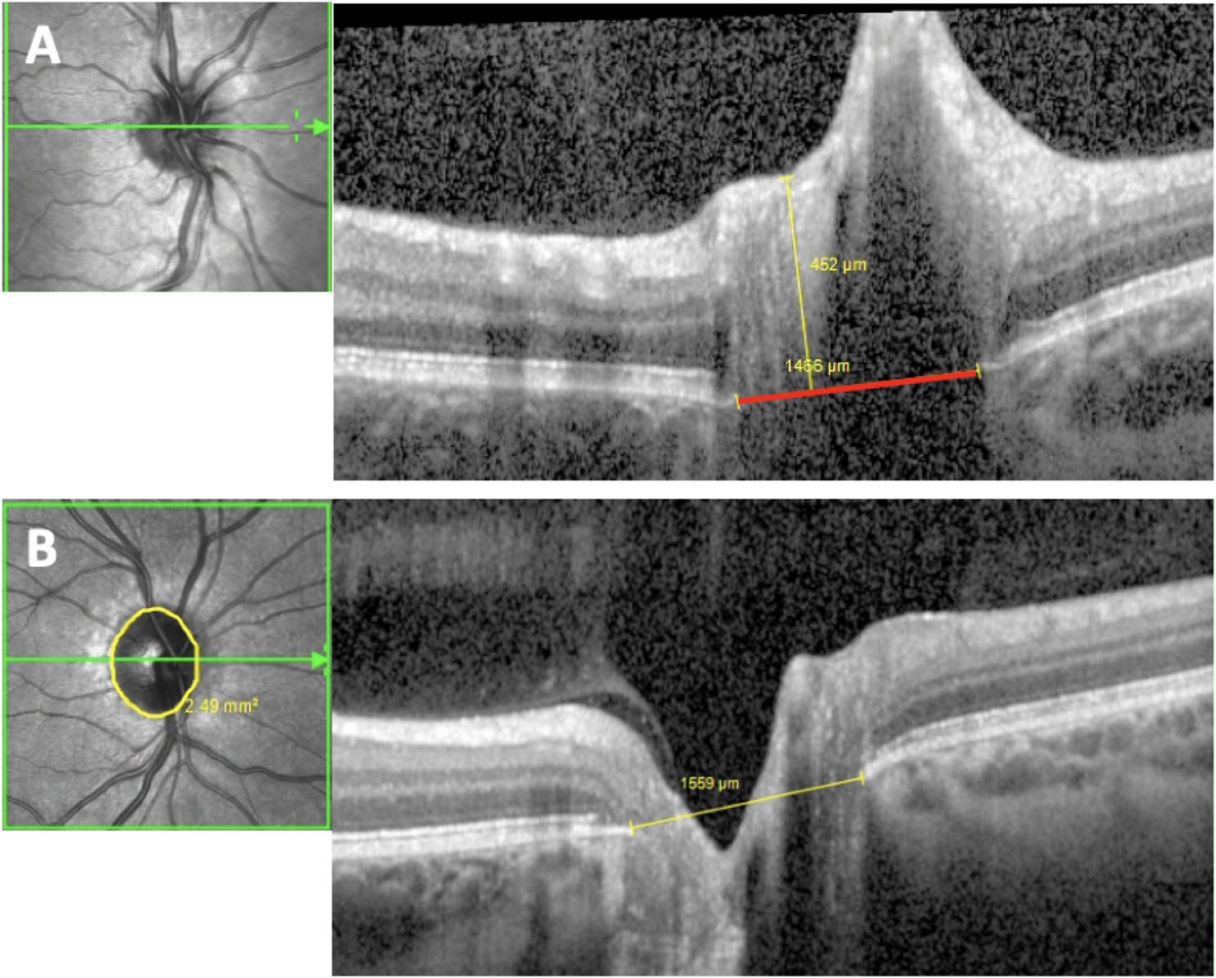
Standardized evaluation of SD-OCT scans of the optic disc. (A) Measurement of prelaminar depth: prelaminar depth is measured as the distance between the internal limiting membrane of the optic nerve head at its lowest point and the Bruch’s membrane opening (BMO) reference plane (BMO reference plane marked in red, prelaminar depth measurement marked in yellow). In this patient the prelaminar depth is positive as tissue is seen above the BMO reference plane. (B) Representative measurement of the optic disc area (yellow circle) and the widest optic disc diameter (yellow line) connecting both ends of the Bruch’s membrane

The prelaminar depth was measured as the distance between the internal limiting membrane of the optic nerve head at its lowest point and the Bruch’s membrane opening (BMO) reference plane (see also Moghimi et al. [15]). The BMO was defined as the termination of the Bruch’s membrane (one point on each side of the B-scan). A positive prelaminar depth signifies that tissue exists above the BMO reference plane (Fig. 1A).

The optic disc area was calculated by the OCT software after manually confining the edges of the optic disc on the infrared image of the optic disc (Fig. 1B).

The line connecting both ends of the Bruch’s membrane horizontally was termed the horizontal disc diameter. The widest disc diameter was used for further investigation (Fig. 1B).

Data of the quantification of corneal cystine crystals using the AS-OCT (anterior segment OCT)-based calliper method and the semiautomated, objective grey-scale based algorithm with a threshold of 211 [16] and data of the quantification of retinochoroidal cystine crystals using the RCCCS (Retinochoroidal Cystine Crystal Score) [7] published in other works from our group were obtained from the previous datasets.

## Blood sampling

Blood samples were collected for the analysis of metabolic parameters (e.g. full blood count, electrolytes, urea, iron levels, thyroid function tests), and determination of cystatin C levels (Institute for Laboratory Medicine, Ludwig-Maximilian University, Munich). To assess the impact of renal function on neuroretinal structures, only the cystatin C levels of patients who had not yet undergone kidney transplantation were included in the calculation. Determination of the white blood cell (WBC) cystine level was performed by the laboratory of the university of Muenster.

### Statistical analysis

All statistical analyses were performed using SPSS Statistics (Version 25.0; IBM; Armonk, New York, USA). First data distribution was assessed visually and statistically. Next, Gausssian data were presented as mean ± SD; ordinal data was presented as median and range. Correlation analyses between the pRNFL thickness, the prelaminar depth, the ONH volume, the mGCL and age, best corrected visual acuity in logMAR, the CCCS, the RCCCS, the depth of penetration of corneal cystine crystals measured by the AS-OCT-based calliper method, the amount of corneal crystal deposition measured by the grey-scale algorithm, the cystatin C levels, the daily cysteamine dose adapted for body weight, the WBC cystine levels or urea were performed by calculating two-sided Pearson’s correlation coefficient. For these tests a Bonferroni correction was applied. A p-value of < 0.05 was considered to indicate statistical significance.

## Results

This study included a total of 40 eyes from 40 patients diagnosed with infantile nephropathic cystinosis. The male to female ratio was 21:19, mean age was 20.6 ± 8.6 (6–37) years and spherical equivalent 0.47 ± 1.85 (-2.63–5.89). Further characteristics of the examined cohort are summarized in Tables 1, 2 and 3. To assess adherence to treatment with cysteamine, a modified composite compliance scoring system (ranging from 0 to 3) was used, as described previously [17]: a score was assigned to each patient for every year of his life; thereafter, a mean individual score was calculated. Specifically, a score of 0 per year was assigned if the patient’s average leucocyte cystine level was ≥ 3 nmol half-cystine/mg protein on cysteamine therapy, or if the patient did not take any cysteamine. A score of 0 was also assigned for every year preceding the diagnosis of infantile nephropathic cystinosis. A score of 1 was attributed if the average leucocyte cystine level was ≥ 2 and < 3 nmol, while a score of 2 or 3 was used if this level was ≥ 1 and < 2 nmol or < 1 nmol, respectively.

The normal control group consisted of 30 eyes from 30 healthy individuals. The male to female ratio was 18:12, mean age was 20.7 ± 12.5 (4–71) years and spherical equivalent 0.47 ± 1.29 (-1.75-4.75). The groups were matched for age (*p* = 0.969) and spherical equivalent (*p* = 0.069, Table 1).

**Table 1:** Demographic and clinical data of the cystinosis group compared to the normal control group

	Cystinosis group *n* = 40 mean ± SD, range	Control group *n* = 30 mean ± SD, range	*p*
No. of eyes (n) No. of patients (n)	40 40	30 30	
Age (y)	20.6 ± 8.6 (6–37)	20.7 ± 12.5 (4–71)	0.969
Age at diagnosis (y)	1.62 ± 1.35 (0.04-8)	-	
Gender (m: f)	21:19	18:12	
Spherical equivalent	0.47 ± 1.85 (-2.63–5.89)	0.47 ± 1.29 (-1.75-4.75)	0.069
Best corrected visual acuity (logMAR)	0.01 ± 0.12 (-0.20-0.30)	-	
Intraocular pressure (mmHg)	14.19 ± 2.83 (9–19)	-	
White blood cell cystine level (nmol/mg protein) (normal range:<1 nmol/mg protein)	0.89 ± 0.72 (0.11–3.20)	-	
White blood cell cystine score	41.89 ± 18.31 (9–91)	-	
eGFR (ml/min/1.73 m²)	56.75 ± 29.66 (17.2-151.8)	-	
Serum cystatin C level (mg/L) (normal range: 0.6–1 mg/L)	2.22 ± 1.50 (1-7.08)	-	
Daily dose of cysteamine (mg/kg body weight/day)	36.92 ± 15.78 (0-66.41)	-	

**Table 2:** Clinical systemic data of all 40 patients of the cystinosis group grouped listed by age

Pat. No.	Age (y)	Mean WBC cystine level	WBC cystine score	eGFR	Serum cystatin C level	CKD stage G/A	Kidney Tx status: Tx (1) No Tx (0)	Type of cysteamine: immediate-release (1) delayed-release (2) combination (3) none (0)	Daily dose of cysteamine
1	6	0.62	12	71	1.22	G2A3	0	2	66.41
2	7	1.04	13	-	0.93	-	0	2	56.3
3	8	0.56	28	35.8	2.74	G3A3	1	1	64.17
4	8	0.52	17	-	0.98	-	0	2	62.25
5	8	0.54	18	119	1	G1A2	0	2	54
6	11	0.53	25	33	2.03	G3A3	0	2	49.86
7	12	0.4	35	151.8	0.86	G1A2	0	3	57.81
8	14	0.82	29	29	2.41	G3A3	0	1	38.63
9	14	0.59	36	60	1.14	G2A3	0	2	35.07
10	14	0.57	35	-	0.69	-	0	2	45.71
11	15	0.42	34	77.6	1.28	G2A3	0	1	52.52
12	15	0.32	29	55.9	1.84	G3A3	1	2	27.32
13	15	0.57	26	60.8	1.69	G2A3	1	2	29.82
14	16	0.53	34	17.7	2.38	G4A3	0	1	40.08
15	16	0.35	20	42.6	2.12	G3A3	0	2	33.87
16	17	0.43	47	68	1.11	G2A3	0	1	40.96
17	17	0.11	48	50.4	1.23	G3A3	0	2	24.47
18	17	0.56	33	65	1.25	G1A3	0	1	32.09
19	18	0.36	48	101	0.86	G1A3	0	2	36.41
20	18	1.5	28	44	0.8	G3A3	0	1	43.8
21	19	0.27	53	55	1.21	G3A3	0	1	42.86
22	19	0.74	9	PD	7.08	PD	0	1	35.29
23	23	0.89	50	34	1.52	G3A3	0	1	29.7
24	24	0.23	69	48	1.99	G3A3	0	1	41.18
25	26	0.92	50	51	1.33	G3A3	1	2	25.71
26	26	2.97	43	HD	5.87	HD	1	0	0
27	26	1.2	44	83	1.39	G2A3	1	2	32.1
28	26	3.2	53	HD	5.15	HD	0	0	0
29	26	0.97	53	17.2	2.35	G4A3	0	1	30.1
30	26	0.41	66	55	1.47	G3A3	1	1	46.1
31	27	0.52	54	94	1.5	G1A2	1	1	33.9
32	28	1.26	56	42	2.29	G3A3	1	1	37.2
33	29	2.4	67	52	-	G3A3	0	1	37.86
34	29	0.98	50	PD	-	PD	1	2	29.6
35	32	1.23	65	27	-	G4A3	1	1	-
36	32	0.37	58	66	1.79	G2A3	1	1	32.2
37	33	1.95	44	52	1.65	G3A3	1	1	12.78
38	33	0.52	91	76	1.18	G2A3	1	1	45.28
39	37	1.95	-	19	4.03	G4A3	1	0	0
40	37	1.23	64	19	3.54	G4A3	1	1	38.1

WBC: white blood cell, CKD: chronic kidney disease, HD: hemodialysis, PD: peritoneal dialysis, tx: transplantation

Mean (over the past two year prior to analysis) WBC cystine level in nmol/mg protein (normal range:<1), eGFR in ml/min/1.73 m² (for children < 17 years estimated by the Schwartz formula; for patients ≥ 17 years estimated by the EPI-CKD formula), Cystatin C in mg/L (normal range: 0.6–1), cysteamine dose in mg/kg body weight/day

**Fig. 2:**
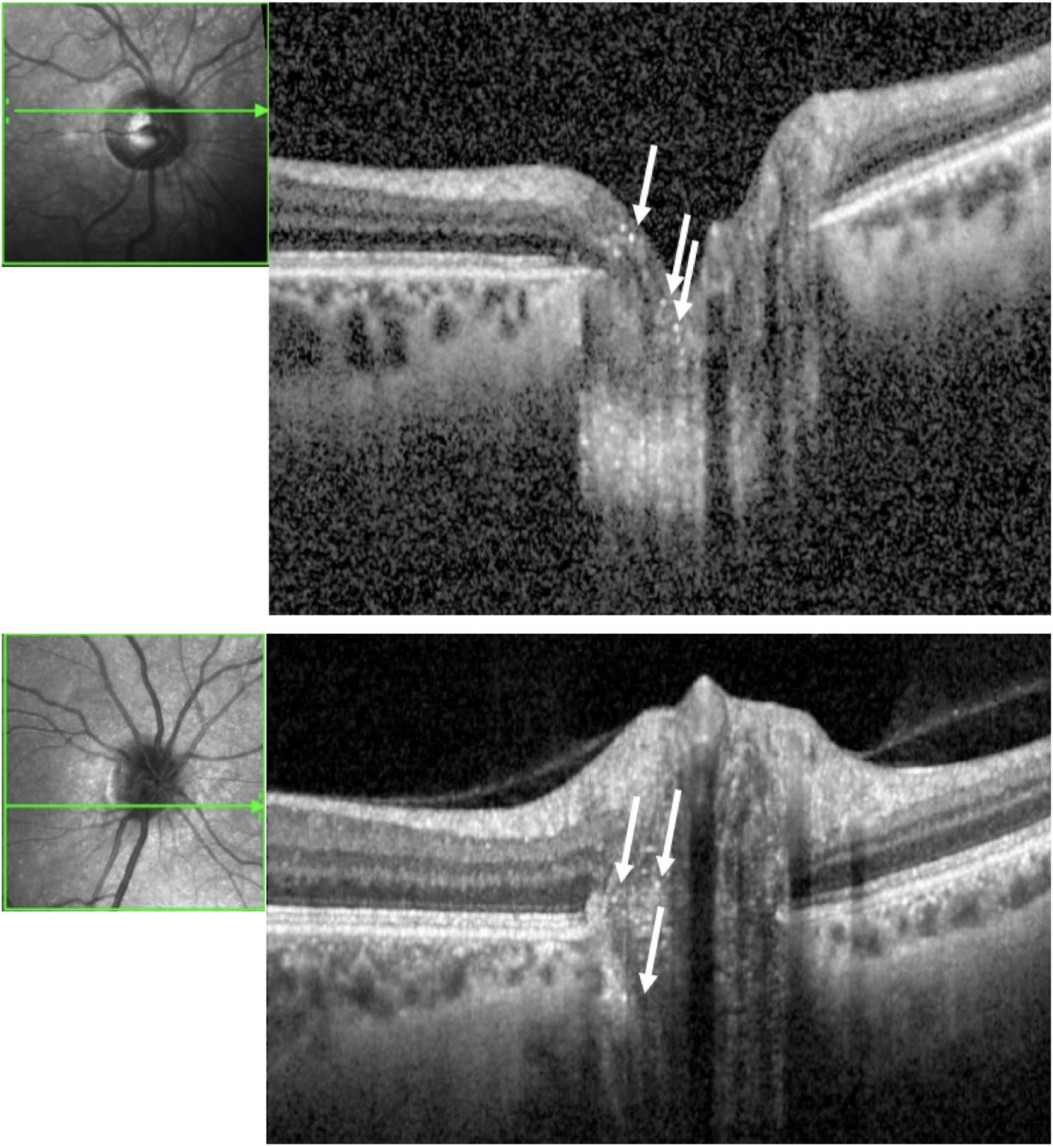
Two exemplary SD-OCT scans of the optic discs from different patients with cystinosis. The hyperreflective cystine crystals (marked with arrows) were found in the optic discs of all subjects scanned

Crystals in the optic nerve and retina are hardly visible by fundoscopy. For this reason, OCT was used. Crystals in the optic nerve head as indicated by hyperreflective foci in the SD-OCT images were detected in all scanned optic nerves (Fig. 2). A closer examination revealed that crystal deposits at the level of the optic disc occur in a high quantity and are already present at an early stage. Deposits follow the course of the retinal nerve fibers in a string-like pattern (Fig. 2).

**Fig. 3:**
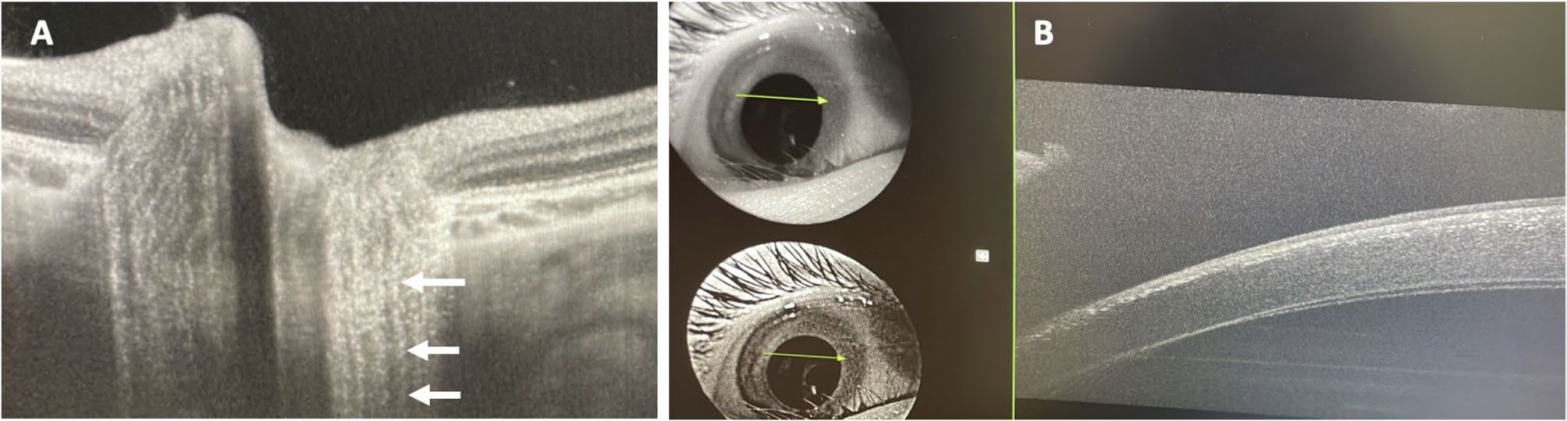
Representative infrared SD-OCT-images of the optic discs of four patients with cystinosis. The en face images show a prominent and crowded appearance of the optic discs

The optic discs in cystinosis patients generally appeared more prominent and crowded (Fig. 3).

## Thicker global pRNFL in cystinosis patients

**Fig. 4:**
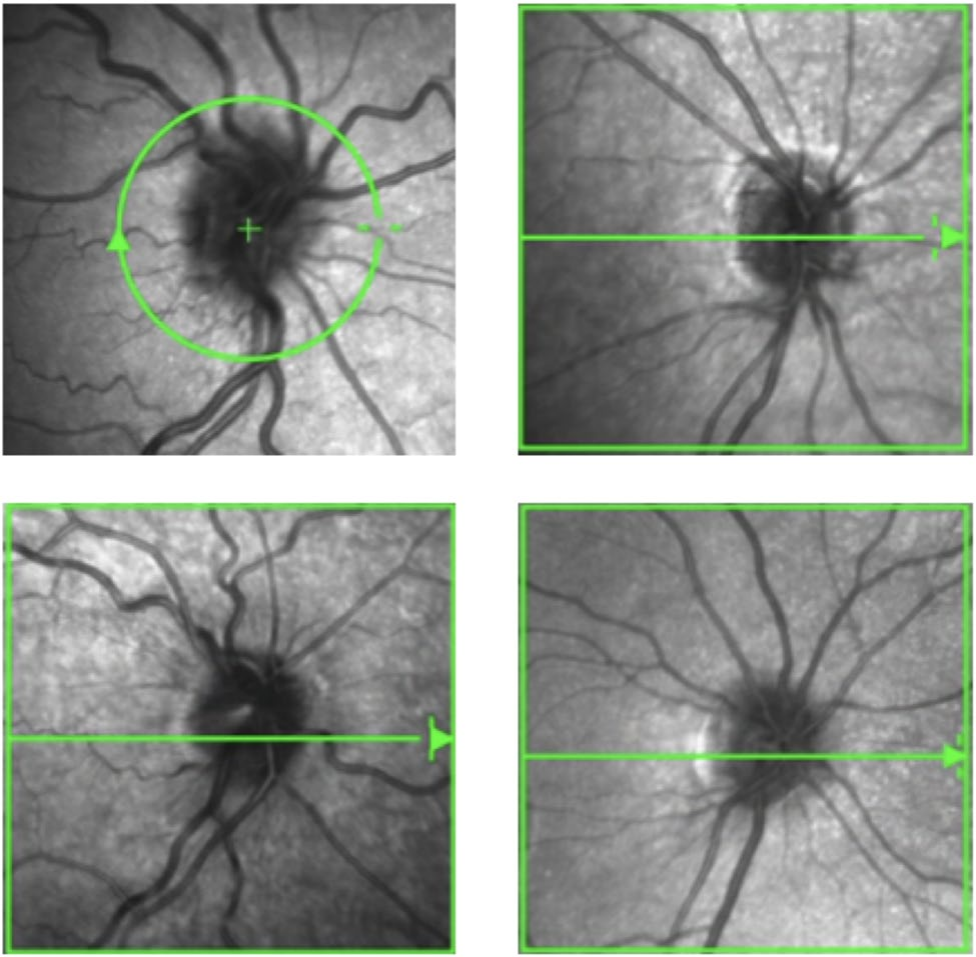
Boxplots showing the median, 25th percentile and 75th percentile of pRNFL thickness (µm) and mGCL volume (mm3, 3 mm diameter) and the significant differences in (A) pRNFL and (B) mGCL between the normal control and the cystinosis group

A significantly thicker global pRNFL was found in the cystinosis group compared to the normal control group, with values of 111.05 ± 20.49 (range 66–205) µm versus 101.27 ± 9.46 (range 85–119) µm (*p* = 0.05, Table 4) (Fig. 4A). Cystatin C levels were elevated in 86.5% of all patients at the time of OCT acquisition with a mean value of 1.96 ± 1.33 (range 0.69–7.08 mg/L, normal values: 0.6-1 mg/L).

In a subgroup analysis of cystinosis patients who showed higher than average pRNFL thickness (16 patients), 81,3% (13 patients) showed a decline in renal function, measured by cystatin C, 50% showed elevated urea levels. In 57.14% anemia was noted, but none of the patients showed an iron deficiency anemia (see Table 3). None of the patients showed laboratory signs of hypocalcemia, hypoparathyroidism, or hypothyroidism, which could be associated with optic disc swelling.

**Table 3:** Laboratory parameters of a cystinosis subgroup of the patients with higher-than-average RNFL thickness

Parameter	Mean ± SD	Range
RNFL thickness (µm)	125.94 ± 22.78	112–205
Urea (mg/dl)	50.56 ± 30.56	20–147
Cystatin C (mg/L) *(Normal range: 0.6-1)*	2.00 ± 1.53	0.86–7.08
Erythrocytes (T/l)	4.38 ± 0.66	3.43–5.82
Hemoglobin (g/dl)	12.61 ± 1.70	10-15.5
MCV (fl.)	85.35 ± 5.61	78–98
MCH (pg)	28.89 ± 2.04	26.4–32.7
Fe (µg/dl)	92.66 ± 40.76	23–172
Ferritin (ng/ml)	222.13 ± 289.6	16–541
Transferrin (g/l)	2.61 ± 0.47	1.4–3.4
TIBC (%)	26.23 ± 12.22	5–61
Calcium (nmol/l)	2.32 ± 0.17	1.93–2.61
TSH µU/ml	1.88 ± 0.72	0.27–3.18
T4 (ng/dl)	1.23 ± 0.29	0.8–1.7
PTH (pg/ml)	106.24 ± 162.79	10.3–886

Global pRNFL thickness showed a tendency to be thicker with increasing depth of penetration of corneal cystine crystals measured by the AS-OCT-based calliper method (*r* = 0.304, *p* = 0.836), but this correlation was not statistically significant. No significant associations were found with increasing age (*r*=-0.103, *p* = 1) or age at first diagnosis (*r* = 0.388, *p* = 1). Also, the pRNFL thickness did not significantly correlate with other clinical parameters, such as best corrected visual acuity in logMAR (*r*=-0.104, *p* = 1), the CCCS (*r*=-0.008, *p* = 1), the amount of corneal crystal deposition measured by the grey-scale algorithm (*r*=-0.270, *p* = 1), the RCCCS (*r* = 0.156, *p* = 0.1), or laboratory parameters such as the cystatin C levels (*r* = 0.126, *p* = 1), the daily cysteamine dose adapted for body weight (*r* = 0.190, *p* = 1), the WBC cystine levels (*r*=-0-041, *p* = 1) or urea (*r* = 0.217, *p* = 1).

## Higher rate of positive prelaminar depth in cystinosis patients

A higher rate of positive prelaminar depth was observed in patients with cystinosis (75% of eyes in the cystinosis group, compared to 30% of eyes in the normal control group; 257.4 ± 190.8 μm vs. 226.7 ± 144.6 μm, Table 4).

The prelaminar depth showed a correlation with increasing RCCCS (*r* = 0.431, *p* = 0.561). No significant associations were found with age (*r* = 0.017, *p* = 1), age at first diagnosis (*r* = 0.215, *p* = 1), clinical parameters, such as best corrected visual acuity in logMAR (*r* = 0.028, *p* = 1), the CCCS (*r* = 0.281, *p* = 1), the depth of penetration of corneal cystine crystals measured by the AS-OCT-based calliper method (*r* = 0.081, *p* = 1), the amount of corneal crystal deposition measured by the grey-scale algorithm (*r* = 0.030, *p* = 1), or laboratory parameters such as the cystatin C levels (*r* = 0.361, *p* = 1), the daily cysteamine dose adapted for body weight (*r*=-0.163, *p* = 1), the WBC cystine levels (*r* = 0.229, *p* = 1) or urea (*r* = 0.160, *p* = 1).

### Small mean optic disc area and cup to disc ratio

Both the cystinosis and the normal control group showed a relatively small optic disc area. The cystinosis group had a mean optic disc area of 1.8 ± 0.38 (range 1.09–2.57) mm², while the normal control group had a mean area of 2.07 ± 0.35 (range 1.21–2.57) mm², *p* = 0.826, Table 4). The cup to disc ratio (CDR) was smaller in the cystinosis group with a mean value of 0.18 ± 0.20 (range 0-0.56), compared to the normal control group with a mean value of 0.34 ± 0.20 (0-0.59), *p* = 0.246, Table 4).

### Slightly larger mean optic nerve head volume in cystinosis patients

The optic nerve head volumes were larger in the cystinosis group (9.2 ± 1.03 (range 7.14–12.34) mm³), as compared to the normal control group (8.6 ± 0.66 (range 6.8–9.8) mm³), but the observed difference was not statistically significant (*p* = 0.254, Table 4).

Correlation analysis revealed that a higher RCCCS was associated with a greater ONH volume (*r* = 0.498, *p* = 0.176). However, ONH volume did not show any significant correlations with age (*r*=-0.187, *p* = 1) or age at first diagnosis (*r* = 0.194, *p* = 1). Nor was ONH volume significantly associated with other clinical parameters, including best corrected visual acuity in logMAR (*r* = 0.103, *p* = 1), the CCCS (*r*=-0.052, *p* = 1), the depth of penetration of corneal cystine crystals measured by the AS-OCT-based calliper method (*r* = 0.250, *p* = 1), the amount of corneal crystal deposition measured by the grey-scale algorithm (*r*=-0.226, *p* = 1), or laboratory parameters such as the cystatin C level (*r* = 0.129, *p* = 1), the daily cysteamine dose adapted for body weight (*r* = 0.299, *p* = 1), the WBC cystine levels (*r* = 0.161, *p* = 1) or urea (*r* = 0.248, *p* = 1).

### Comparable widest horizontal disc diameter

The widest horizontal disc diameters were comparable between the two groups (1608.24 ± 207.48 (range 1044–2011) µm in the cystinosis group versus 1587.93 ± 158.45 (range 1328–2012) mm² in the normal control group, *p* = 0.674, Table 4).

### Smaller mGCL volume in cystinosis patients

Regarding the mGCL volume, a significantly smaller volume was found in the cystinosis group as compared to the normal controls with a mean of 0.25 ± 0.03 (range 0.17–0.36) mm³ in the cystinosis group and 0.35 ± 0.03 (range 0.29–0.41) mm³ in the normal control group (*p* = 0.036, Fig. 4B; Table 4).

Correlation analysis revealed that mGCL volume did not show any significant association with increasing age (*r*=-0.162, *p* = 1) or age at first diagnosis (*r*=-0.211, *p* = 1). Additionally, it did not significantly correlate with clinical parameters, such as best corrected visual acuity in logMAR (*r*=-0.295, *p* = 0.715), the CCCS (*r* = 0.136, *p* = 1), the depth of penetration of corneal cystine crystals measured by the AS-OCT-based calliper method (*r* = 0.019, *p* = 1), the amount of corneal crystal deposition measured by the grey-scale algorithm (*r* = 0.055, *p* = 1), the RCCCS (*r* = 0.036, *p* = 1), or laboratory parameters such as the cystatin C levels (*r*=-0.149, *p* = 1), the daily cysteamine dose adapted for body weight (*r* = 0.168, *p* = 1), the WBC cystine levels (*r* = 0.122, *p* = 1) or urea (*r* = 0.301, *p* = 1).

**Table 4:** Optical coherence tomograpy parameters of the optic disc in the cystinosis group compared to the normal controls

	Cystinosis group mean ± SD, range	Control group mean ± SD, range	*p*
pRNFL (µm)	111.05 ± 20.49 (66–205)	101.27 ± 9.46 (85–119)	0.05
mGCL (mm³)	0.25 ± 0.03 (0.17–0.36)	0.35 ± 0.03 (0.29–0.41)	0.04
Positive prelaminar depth (% of eyes)	75	30	0.00
Optic disc volume (mm³)	9.2 ± 1.03 (7.14–12.34)	8.6 ± 0.66 (6.8–9.8)	0.25
Optic disc area (mm²)	1.8 ± 0.38 (1.09–2.57)	2.07 ± 0.35 (1.21–2.57)	0.83
Cup to disc ratio	0.18 ± 0.20 (0-0.56)	0.34 ± 0.20 (0-0.59)	0.25
Widest horizontal disc diameter (µm)	1608.24 ± 207.48 (1044–2011)	1587.93 ± 158.45 (1328–2012)	0.68

## Discussion

**Fig. 5:**
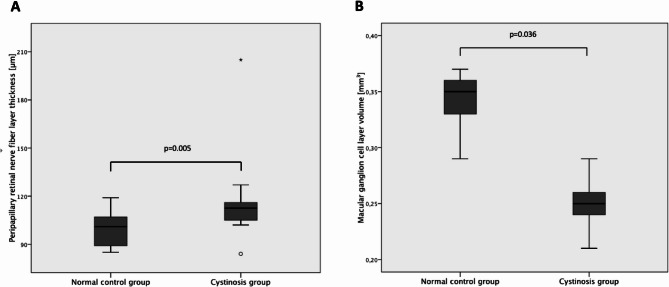
Swept source OCT of the optic disc (A) and the cornea (B) of a 2-year-old cystinosis patient from our clinic. Already early, distinct cystine crystal deposition can be seen in the area of the optic nerve head (A, marked with arrows). Deposits follow the course of the nerve fibers. Only very few crystals were visible in the cornea at this time (B)

The present study provides novel evidence of pronounced and early cystine crystal accumulation in the optic nerve heads of patients with infantile nephropathic cystinosis. The deposition can occur concomitantly with the well-described cystine crystal deposits in the cornea and seems to appear in high quantities (Figs. 2 and 3). In Fig. 5 representative images of a 2-year-old cystinosis patient showing distinct crystal accumulation in the optic disc with very few corneal cystine crystals are shown. Crystal deposits follow the course of the retinal nerve fibers in a string-like pattern (Figs. 2 and 3). Moreover, the deposition is observed prior to the accumulation of cystine deposits in the choroid and retina; a previous study from our group using the same collective found that only 68.2% of all scanned cystinosis patients showed retinochoroidal cystine crystal deposits in a comparatively low quantity [7].

This is an entirely new and significant aspect in the search for the pathogenesis of cystinosis.

After deep phenotyping of a large cystinosis cohort, it was noticed that the optic discs in these individuals tend to be more prominent exhibiting a significantly thicker RNFL and a higher rate of positive prelaminar depth (Figs. 1A, 4 and 5A) as compared to a normal control group.

So far, only a few case series have reported changes in the optic nerve heads of cystinosis patients, primarily focusing on papilledema due to raised intracranial pressure [18, 19, 20]. This may be attributed to cystine deposits in the arachnoid villi and meninges, inhibiting outflow of cerebrospinal fluid [19]. In infantile nephropathic cystinosis, various factors may contribute to the development of secondary pseudotumor cerebri, including renal failure, electrolyte imbalances, treatment with growth hormone, thyroxine, calcineurin inhibitors, cysteamine itself or the tapering of steroids [21]. However, in our cohort ONH volumes that are used to quantify real papilledema and are correlated with the Frisén scale grading [22, 23] were comparable between the two study groups. The appearance was more consistent with a prominent optic disc with a higher rate of positive prelaminar depth. The underlying etiology of these more prominent, almost crowded discs remains to be elucidated; SD-OCT imaging clearly demonstrated that the cystine crystals follow the course of the nerve fibers in a string-like pattern (Figs. 2 and 3).

Nerve fibers are segregated into about 1000 bundles, or fascicles, by astrocytes. In the external part of the lamina cribrosa, columns predominantly consisting of oligodendrocytes with a few astrocytes, are interspersed between the nerve fibers [24]. While OCT imaging can only assist in forming hypotheses on the precise cellular origin of cystine crystals, the string-like appearance may point towards a neuroglial localization. Furthermore, glial dysfunction contributing to neurodegeneration, is commonly observed in various other lysosomal storage diseases, since glial cells play a critical role in lysosomal homeostasis [25].

In a previous study conducted by our group, retinal crystals were found to be located predominantly in the GCL and inner nuclear layer at the transition zones with the adjacent inner -and outer plexiform layer. These transition zones coincide with the regions containing the retinal microvasculature [7]. Retinal microglia are also located in the ganglion cell layer, both plexiform layers, and specifically around the vessels of the superior retinal vascular plexus [26]. This suggests that our previous morphological evaluations of both the retina and the optic nerve may indicate crystal deposition, particularly in the glial cells of the retina or the optic nerve. In a recent work by Üzüm et al. using in vivo confocal biomicroscopy, crystal deposition in the cornea was also described as being present specifically in the subepithelial nerve plexus [27]. Histological work by Vogel et al. identified crystal deposition within the oligodendrocytes and pericytes of the brain [28].

All in all, even if the findings point towards a primarily glial deposition of the cystine crystals, the question of the exact localization still needs to be assessed further. Histological studies are necessary to accurately differentiate the perivascular cell types involved, such as glial cells, pericytes, and endothelial cells [29].

The structural investigations of the optic discs revealed that cystine crystals are deposited in a high quantity and appear already at an early stage of the disease (Figs. 2 and 3). The optic discs in patients with cystinosis are generally more prominent, as seen by a significantly thicker RNFL and higher rate of positive prelaminar depth (Fig. 3).

Case reports described uremic optic neuropathy with optic disc swelling as a rare complication of high urea levels associated with kidney dysfunction. However, in these patients, a significant decline in visual acuity was reported [30, 31, 32]. In contrast, none of the patients in the present cohort exhibited a significant decline in visual acuity (Table 1). In addition, iron deficiency anemia which can cause a hyper viscous state and is associated with papilledema due to elevated intracranial pressure, has been reported as a potential cause of optic disc swelling [33]. In our cohort, none of the patients showed signs of iron deficiency anemia and the optic discs displayed a prominent, crowded appearance.

These findings suggest that the observed disc changes may be attributed to local crowding caused by distinct cystine crystal accumulation at the level of the optic disc: The significant accumulation of cystine crystals at the optic nerve head may lead to axoplasmic stasis and a decline in function of oligodendrocytes and astrocytes with consequent inhibited glutamate signaling [34]. Axoplasmic stasis and local inflammatory processes may cause the observed slight ONH crowding.

Oligodendrocytes are exceptionally sensitive to any insults to the CNS, such as injury, ischemia, or inflammation, which ultimately results in the loss of oligodendrocytes and myelin, and eventually secondary axon degeneration [35]. This could help explain the secondary GCL thinning (Fig. 4B) observed in cystinosis patients, alongside metabolic factors, such as changes from kidney dysfunction. Several large-scale studies [36, 37] reported circumpapillary thinning of the pRNFL and mGCL in patients with a decline in kidney function possibly due to a dysregulation of the renin-angiotension system, which is present in both the retina and the kidneys, with associated retinal microvascular damage or oxidative stress. Despite a high number of patients (86.5%) showing elevated cystatin C levels as a marker for kidney dysfunction, our cohort demonstrated significant thickening of the pRNFL, supporting the hypothesis that local factors, such as the distinct crystal deposition in the optic nerve, could play an important role. Furthermore, ONH volume was greater and prelaminar depth showed a tendency to be higher in patients with higher RCCCS values, indicating that a higher load of cystine crystals in neuroretinal and retinal structures may be linked with increased crowding.

This is the first large-scale evaluation of cystinosis patients to examine for glaucomatous changes. To date only few case reports exist reporting glaucoma due to pupillary-block [38]. Glaucomatous optic atrophy or elevated intraocular pressure could not be found in any patient of our cohort.

One limitation of this study is the inability to quantify crystals at the level of the optic disc due to their hyperreflectivity and their very condensed deposition. In many cases, precise demarcation was therefore not feasible. However, with advances in deep learning based retinal OCT segmentation, objective and fully-automated scoring of crystals at the level of the optic disc may be possible in the near future [39]. Similarly, we were unable to perform OCT scans on all very young patients due to lack of compliance. Future studies could address this limitation using handheld OCT devices [40]. A notable strength of this study is its relatively large sample of 40 eyes. Infantile nephropathic cystinosis has an estimated incidence of 1:100.000–1:200.000 [41]. Therefore, the cohort of cystinosis patients presented in this study may be regarded as highly valuable in broadening our understanding of this rare genetic disorder.

## Conclusions

Based on the findings outlined above, we conclude that a pronounced cystine crystal deposition at the level of the optic disc can be seen in all scanned optic discs. This accumulation occurs in substantial amounts and in concomitance with the corneal manifestations that were previously considered to be the foremost ocular sign of cystinosis.

Crystals follow the optic nerve fibers in a string-like pattern, making a primary deposition in glial cells possible. Moreover, a plurality of findings from the present study indicates that the optic discs of cystinosis patients appear to be crowded (significantly thicker RNFL compared to the normal controls, higher rate of positive prelaminar depth). Whether this crowding is caused by local compression and axoplasmic stasis due to a pronounced crystal accumulation has yet to be determined. The generalized GCL thinning may be caused by kidney dysfunction. There are no signs of glaucomatous defects in any of the patients. Future studies are needed to further examine the complex interactions of this multi-system disorder.

## Data Availability

The datasets used and analysed during the current study are available from the corresponding author on reasonable request.
